# Long-Term Effects of Maternal Subnutrition in Early Pregnancy on Cow-Calf Performance, Immunological and Physiological Profiles during the Next Lactation

**DOI:** 10.3390/ani9110936

**Published:** 2019-11-08

**Authors:** Agustí Noya, Isabel Casasús, Javier Ferrer, Albina Sanz

**Affiliations:** Department of Animal Production, Centro de Investigación y Tecnología Agroalimentaria (CITA) de Aragón, Instituto Agroalimentario de Aragón, IA2 (CITA-Universidad de Zaragoza), 50059 Zaragoza, Spain; anoya@cita-aragon.es (A.N.); icasasus@cita-aragon.es (I.C.); jferrerac@aragon.es (J.F.)

**Keywords:** prenatal undernutrition, beef cattle, colostrum, passive transfer immunity, IgG, IgM, IGF-1

## Abstract

**Simple Summary:**

Early pregnancy is a crucial stage in the fetus development. In this phase, from undifferentiated cells, equal to each other, tissues and organs start to develop. Nutrition and metabolic status of the cow during pregnancy affect the intrauterine environment and the nutrient source for the fetus. Therefore, cow diet during early pregnancy affects the fetus development and could have long-term consequences on the future calf. In this study, we assessed the effects of a poor maternal diet during the first third of gestation on the performance of cows and calves during the next lactation, as well as the effect on the transfer of immunity from cow to calf. We used Parda de Montaña and Pirenaica cow-calf pairs, two Spanish autochthonous beef breeds. We concluded that maternal undernutrition reduced the body fat reserves of cows at calving, which affected most of the cow productive parameters and the colostrum immunoglobulin concentration. Furthermore, poor maternal diet altered the calf development and metabolic status, with reduced size and weight at weaning, especially in the Pirenaica breed, which prioritized the cow maintenance instead of the calf growth.

**Abstract:**

This study aimed to evaluate the effects of undernutrition during the first third of gestation on cow-calf performance, immunological and physiological profiles during the next lactation in two cattle breeds. Fifty-three Parda de Montaña (PA) and 32 Pirenaica (PI) cows were inseminated, assigned to one of two diets (CONTROL or SUBNUT; 100% or 65% of their requirements) until day 82 of gestation, and fed 100% of the requirements during gestation and next lactation. Cow and calf performance were assessed during lactation. Colostrum and cow-calf plasma samples were analyzed to assess the passive transfer of immunoglobulins and to characterize energy metabolism. At calving, SUBNUT cows had a lower body condition score, which impaired most of the cow-calf parameters. All cows had considerable weight losses during lactation except for SUBNUT-PI cows. Colostrum immunoglobulin G (IgG) concentration was lower in SUBNUT-PI cows, and milk fat content was higher in SUBNUT cows. SUBNUT calves had lower values of body measurements at weaning, and calves born from SUBNUT-PI dams had lower milk intake and the lowest average daily gain (ADG), which was reflected in their lower plasma insulin-like growth factor-1 (IGF-1) concentration. In conclusion, undernutrition in early gestation in suckler cows had long-term effects on offspring postnatal growth, this physiological evidence being more severe in Pirenaica cow-calf pairs.

## 1. Introduction

Undernutrition periods are frequent in beef cattle production systems. To reduce feed costs, cattle have had to adapt to low-cost diets or to extensive management, where animal intake depends mostly on food availability. A poor diet because of low quality or low quantity affects the cow production cycle, for example, the peri-implantational period and early gestation. The impact of subnutrition and a poor uterine environment on the embryo/fetus during the first stages of gestation are not fully understood. Long et al. [1] described no differences in calf birth weight in underfed dams during early gestation, whereas Micke et al. [2] reported reduced calf birth weight in nutrient restricted heifers during the first two thirds of gestation. The peri-implantational period is a vulnerable phase, during which adverse programming mediated through poor maternal nutrition might begin [3,4,5]. Under adverse intrauterine conditions, the fetus could permanently modify some endocrine functions to ensure its survival [6]. The structure, physiology and metabolism of different organs and systems could be modified, leading to detrimental postnatal metabolic changes [7] and predisposing offspring to cardiovascular, metabolic and endocrine diseases in later life [8]. Cow energy status during gestation could also have an effect on the adaptive immune response of their progeny. Different studies evaluated the effect of restricted maternal diet during mid [9] or late [10,11] gestation on passive immunity of beef cattle; however, little is known about the effects of maternal feed restriction in early pregnancy. 

Furthermore, these effects could vary depending on the genetic background. Differences in the response to different feeding management have been reported between Parda de Montaña (PA) and Pirenaica (PI) breeds [12,13], which are the two main beef cattle breeds adapted to a semi-extensive system of animal husbandry in the Pyrenees mountain region (northern Spain). 

Our hypothesis was that a poor nutritional diet during early pregnancy could adversely impact the passive transfer of immunity from dam to calf, and offspring physiology and growth during its postnatal life, and this effect would be modulated by cow genotype. Therefore, this study aimed to analyze the effects of undernutrition during the first third of gestation on cow-calf performance and their immunological, metabolic and endocrine profiles throughout the following lactation in PA and PI cow-calf pairs.

## 2. Materials and Methods 

All procedures were approved by the Animal Ethics Committee of the Centro de Investigación y Tecnología Agroalimentaria (CITA) de Aragón. The care and use of animals were performed in accordance with the guidelines of the European Union (Directive 2010/63/E.U.) regarding the protection of animals used for experimental and other scientific purposes [14].

### 2.1. Animals, Management and Diets

This study was conducted at La Garcipollera Research Station, in the mountain area of the Pyrenees (northeastern Spain, 945 m a.s.l.), from December 2014 to June 2016. Fifty-three PA and 32 PI multiparous cows were synchronized to estrus with a protocol based on a progesterone-releasing intravaginal device (PRID Delta 1.55 g, CEVA, Loudéac, France) and a 10 μg injection of gonadotropin-releasing hormone (GnRH, Busol, INVESA, Barcelona, Spain), followed 7 days later by 150 μg of prostaglandin F2α (Galapán, INVESA, Barcelona, Spain). The PRID was removed after 9 days, and a 500 IU injection of pregnant mare serum gonadotropin (Serigan, Laboratorios Ovejero, León, Spain) was administered. A second 10 μg injection of GnRH was administered 48 h later. Eight hours after the second GnRH injection, cows were randomly inseminated with sires of proven fertility (4 PA males for the PA cows and 3 PI males for the PI cows) by an expert technician. After fixed-time artificial insemination (AI), cows were distributed into 2 nutritional treatments and fed individually with a total mixed ration (alfalfa hay, 25.0%; cereal straw, 25.0%; crushed barley, 25.0%; dehydrated alfalfa, 10.0%; rapeseed meal, 6.5%; citrus pulp, 4.5%; soybean meal, 2.5%; and vitamin-mineral complex, 1.5%; Table 1) during the first 82 days of pregnancy. The control group (CONTROL, *n* = 37, 574 ± 8.9 kg live weight (LW); 2.80 ± 0.038 body condition score (BCS) on a 5-point scale) fed a diet that supplied 100% of the estimated energy requirements for cow maintenance, lactation and gestation (10.9 and 10.0 kg DM/cow/d for PA and PI, respectively); and the nutrient-restricted group (SUBNUT, *n* = 48, 568 ± 7.6 kg LW; 2.86 ± 0.032 BCS) received 65% of their requirements (7.0 and 6.4 kg DM/cow/d for PA and PI, respectively), calculated for a 580 kg beef cow producing 9 kg (PA) or 8 kg (PI) of energy-corrected milk [15]. After this treatment phase, the CONTROL group maintained its LW and BCS (583 ± 8.6 kg LW; 2.90 ± 0.040 BCS), whereas they decreased in SUBNUT animals (538 ± 7.2 kg LW; 2.65 ± 0.033 BCS, *p* < 0.001) [16]. After the first third of gestation, all dams were fed 100% of the requirements during the remainder of gestation and the next lactation, using the same total mixed ration described above. Feed was provided at 8:00 and cows were tied up for maximum 2 h until they finished the restricted amount assigned to each one. During lactation, suckling offspring had a restricted twice-daily nursing system, comprising two 30 min periods at 7:00 and 14:30. Their diets only consisted of colostrum and milk from their respective mothers. Calves were weaned at the age of 120 days.

### 2.2. Cow and Calf Performance

Cows and calves were weighed fortnightly to calculate by linear regression the average daily gain (ADG) during lactation. The cow BCS was assessed at calving, in the middle and at the end of lactation by 2 expert technicians, based on the estimation of fat covering the loin, ribs and tailhead [17]. Morphometric measurements of calves were assessed on d 7 and 120 after birth. The variables were height at withers (distance from the floor to the highest point of the withers), height at rump (distance from the floor to the highest point of the internal tuberosity of ilium), rump width (maximum distance between iliac tuberosities), rump length (distance from the ischial tuberosity to the external iliac tuberosity), body length (distance from the cranial side of the shoulder blades to the caudal side of the ischial tuberosity), and heart girth (circumference immediately behind the shoulder blades in a plane perpendicular to the body axis). Cow samples for progesterone plasma analyses were collected into heparinized tubes (BD Vacutainer Becton-Dickenson and Company, Plymouth, UK) twice per week during lactation. Blood samples were centrifuged at 3500 rpm for 20 min at 4 °C immediately after collection. Plasma samples were harvested and frozen at −20 °C until analysis. Plasma progesterone concentration (ELISA test, sensitivity: 0.27 ng/mL) was measured using a specific kit for cattle (Ridgeway Science, Lydney, UK). The mean intra-assay and inter-assay coefficients of variation were 8.0% and 10.4%, respectively. The onset of luteal activity in cows after calving was considered when progesterone concentration was >1 ng/mL. If cows had not ovulated prior to the end of lactation (d 120), the interval to first ovulation after calving was regarded as this date [18].

### 2.3. Colostrum and Milk Composition, Milk Yield and Calf Milk Intake

Colostrum samples were manually collected twice: in Period 1 (from 0 to 12 h postpartum) and in Period 2 (from 12 to 24 h postpartum). Samples from each udder quarter were pooled. On d 23 postpartum, milk yield was recorded by the oxytocin and machine milking method 6 h after calf removal [19]. Fat, protein, lactose and somatic cell count of colostrum and milk were analyzed with an infrared scan (Milkoscan 4000; Foss Electric Ltd., Hillerød, Denmark). Milk data for fat and protein content were used to calculate energy-corrected milk (ECM) yield (adjusted to 3.5% fat and 3.2% protein [20]).

On d 25 and 120 postpartum, calf milk intake was estimated by the weigh–suckle–weigh method. Calves were weighed before and after one 30 min suckling period each in the morning (7:00) and afternoon (14:30). The calf daily milk intake was the sum of the weight differences of the 2 suckling periods (adapted from Rodrigues et al. [21] and Shee et al. [22]).

### 2.4. Immunoglobulin Concentration in Colostrum and Cow-Calf Plasma

To determine plasma immunoglobulin (Ig) G and IgM concentration in cows, blood samples were collected into EDTA tubes (BD Vacutainer Becton-Dickenson and Company, Plymouth, UK) at d 253 post AI (approximately 1 month before calving) and at calving. Colostrum samples were collected at Period 1 (0–12 h postpartum) and Period 2 (12–24 h postpartum), and calf blood samples were taken 48 h after birth (adapted from McGee et al. [11]). Blood samples were centrifuged at 3500 rpm for 20 min at 4 °C and plasma and colostrum samples were frozen at −20 °C until analysis. Concentration of IgG and IgM (ELISA bovine test, sensitivity: 4.8 ng/mL for IgG and 4.5 ng/mL for IgM) were determined using a specific bovine kit (Bovine IgG ELISA Quantitation Set, Cat.No. E10-118; and Bovine IgM ELISA Quantitation Set, Cat.No. E10-101; Bethyl, Montgomery, TX, USA). Plasma samples were diluted at 1:300,000 and 1:20,000 for the IgG and IgM analysis, respectively, and colostrum samples were diluted at 1:500,000 and 1:50,000 for the IgG and IgM analysis, respectively. ELISA tests were carried out according to the manufacturer’s guidelines. To reduce nonspecific binding, 1:9 diluted gelatin from coldwater fish skin (No. G7765, Sigma-Aldrich, St Louis, MO, USA) was added to the set blocking solution. Low binding tubes (Protein LoBind tube 2.0 ml, Eppendorf, Hamburg, Germany) were used to minimize protein sample loss. The mean intra- assay and inter-assay coefficients of variation were 3.2% and 5.5% for IgG, and 2.5% and 2.7% for IgM, respectively.

### 2.5. Metabolic and Endocrine Profiles of Cows and Calves

Blood samples were collected monthly into EDTA or heparinized tubes by coccygeal (in cows) or by jugular (in calves) venipuncture. Furthermore, in the case of IGF-1, blood samples were previously taken every month from cows during the first third of gestation (when the maternal nutritional treatment was applied). Blood samples were centrifuged at 3500 rpm at 4 °C, and plasma samples were taken and frozen at −20 °C until analysis. An automatic analyzer (GernonStar, RAL/TRANSASIA, Dabhel, India) was used to measure blood concentration of glucose (glucose oxidase/peroxidase method, sensitivity: 0.056 mmol/L) and urea (kinetic UV test, sensitivity: 0.170 mmol/L). The mean intra-assay and inter-assay coefficients of variation for these molecules were <5.4% and <5.8%, respectively. Non-esterified fatty acids (NEFA, enzymatic method, sensitivity: 0.06 mmol/L) were analyzed using a commercial kit (Randox Laboratories Ltd., Crumlin Co., Antrim, UK). The mean intra- and inter-assay coefficients of variation were 5.1% and 7.4%, respectively. Insulin-like growth factor 1 (IGF-1, enzyme immunoassay, sensitivity: 20 ng/mL) was determined using a solid-phase enzyme-labeled chemiluminescent immunometric assay (Immulite, Siemens Medical Solutions Diagnostics Limited, Llanberis, Gwynedd, UK). The mean intra-assay and inter-assay coefficients of variation were 3.1% and 12.0%, respectively.

### 2.6. Statistical Analyses

All statistics were calculated using the SAS statistical package v 9.4 (SAS Institute Inc., Cary, NC, USA). The normal distribution of data was assessed with the Shapiro-Wilk test (*p* > 0.05). Normality could not be confirmed for somatic cell count and postpartum anoestrus length; therefore, their values were expressed as a decimal logarithm for further analyses. The ADG of dams and calves, milk chemical composition, postpartum anoestrus length and Ig plasma concentration in calves were analyzed with a generalized linear model (GLM procedure) with the nutritional treatment (CONTROL vs. SUBNUT), breed (PA vs. PI) and their interaction as fixed effects. The cow BCS at calving was included as a covariate. The cow BCS, cow and calf LW, colostrum chemical composition, cow plasma and colostrum Ig concentration, morphometric measurements in calves, nutritional metabolites (glucose, NEFA and urea) and hormone (IGF-1 and progesterone) concentration were analyzed using a mixed linear model (MIXED procedure) for repeated measures based on Kenward–Roger’s adjusted degrees of freedom solution. The fixed factors were nutritional treatment, breed, and their interactions as the between-subject effects, sampling day as the within-subject effect, animal as the random effect (experimental unit), and the cow BCS at calving was included as a covariate. The least square (LS) means of the treatments were estimated per fixed effect, and pair-wise comparisons of the means were obtained by the probability of difference (PDIFF) option of the LS means procedure. Association between nutritional treatment or breed with the cow luteal activity was assessed using the F-test (FREQ procedure). The relationship between metabolite and hormone concentration was determined through Pearson’s correlation coefficients. The level of significance for all tests was *p* < 0.05. Results are presented as LS means ± standard error. 

## 3. Results

### 3.1. Cow and Calf Performance

Nutritional treatment in early gestation affected cow BCS at calving (Table 2), and CONTROL cows had higher BCS than SUBNUT cows (*p* = 0.032); however, these differences disappeared at mid-lactation and at weaning (*p* > 0.05). Regarding the breed, PI cows had higher BCS than PA cows (*p* < 0.001) during all lactation.

Cow LW was not influenced by the nutritional treatment, breed or time (*p* > 0.05, Table 2). The calf LW was affected by a triple interaction among the nutritional treatment, breed and time (*p* = 0.006, Table 3). The PA calves (CONTROL and SUBNUT) were heavier at birth than their PI counterparts. At weaning, no differences were found among PA and CONTROL-PI calves, and SUBNUT-PI calves were the lightest (*p* < 0.001), namely, 29 kg less than their CONTROL-PI counterparts.

Concerning ADG, an interaction between nutritional treatment and breed was observed in cows and calves (Table 3). During lactation, all groups had LW losses, except for those of SUBNUT-PI cows, which were negligible. Contrarily, SUBNUT-PI calves had the lowest ADG (*p* = 0.042). A negative correlation was found between ADG of cows and calves (*r* = −0.37, *p* < 0.001). The cow BCS at calving positively influenced the calf ADG during lactation (*r* = 0.30, *p* = 0.006), with 0.25 kg/d extra for each extra BCS point.

Nutritional treatment in early gestation had no effects on any morphometric parameters on d 7 of life (Table 4). On d 120, height at withers (*p* = 0.046), rump and body length (*p* = 0.020 and *p* = 0.022, respectively) and heart girth (*p* = 0.004) were higher in the CONTROL than in SUBNUT calves. The breed affected most of the morphometric traits because PA calves had higher values than PI calves on d 7 and 120 in height at withers (*p* = 0.001 and *p* < 0.001, respectively) and heart girth (*p* < 0.001 and *p* = 0.024, respectively), on d 7 in rump width (*p* = 0.045) and on d 120 in height at rump (*p* = 0.002). The heart girth was positively influenced by the cow BCS at calving (*p* < 0.001), with 8.2 cm extra for each extra BCS point.

Four months after calving, 12 cows did not have any luteal activity, with no nutritional treatment (8% vs. 19% for CONTROL and SUBNUT, respectively, *p* > 0.05) or breed effect (19% vs. 7% for PA and PI, respectively, *p* > 0.05). No differences were found in postpartum anoestrus length between CONTROL and SUBNUT cows (40 vs. 46 days, respectively, *p* > 0.05). Pirenaica cows needed fewer days to recover the luteal activity than PA (38 vs. 49 days, respectively, *p* = 0.035). The BCS at calving was correlated with the postpartum anoestrus length (*r* = −0.47, *p* < 0.001).

### 3.2. Colostrum and Milk Composition, Milk Yield and Calf Milk Intake

Colostrum chemical composition was not influenced by the nutritional treatment (*p* > 0.05, Table 5). The breed had a significant effect because colostrum lactose content was higher (*p* = 0.015) and somatic cell count was lower (*p* = 0.043) in PI than in PA cows in those samples collected in Period 1 (0–12 h postpartum). Regarding the time when the colostrum sample was taken, the protein concentration decreased (*p* < 0.001) and the lactose concentration increased (*p* < 0.001) from Period 1 to Period 2 (12–24 h postpartum).

Nutritional treatment did not affect milk yield on d 23 but influenced milk chemical composition (Table 6). SUBNUT dams had higher milk fat concentration than CONTROL dams (*p* = 0.010). The breed affected most milk traits, with higher milk yield and somatic cell counts (*p* = 0.008 and *p* = 0.002, respectively), and lower fat and lactose concentration (*p* < 0.001) in PA than in PI cows. No interaction between nutritional treatment and breed was found in milk traits, and therefore, milk yield on d 23 did not differ within breed between the two nutritional treatments (CONTROL-PA, 10.6 kg/d; SUBNUT-PA, 9.2 kg/d; CONTROL-PI, 8.4 kg/d; SUBNUT-PI, 8.2 kg/d; standard error of the mean, 0.23 kg/d). The cow BCS at calving was correlated with milk protein concentration (*r* = 0.29, *p* = 0.008) and ECM yield (*r* = 0.24, *p* = 0.030).

The fat and lactose concentration from colostrum to milk on d 23 increased approximately 35.3% and 52.6%, respectively, and the protein concentration and somatic cell counts decreased approximately 79.6% and 83.2%, respectively.

Regarding the calf milk intake, an interaction was found between nutritional treatment and breed. SUBNUT-PI calves had the lowest milk intake on d 25 (*p* < 0.001), whereas no differences were found among CONTROL-PI and PA calves (*p* > 0.05). On d 120, differences between PI groups disappeared, and PA calves had higher intake than PI calves (*p* < 0.001). Calf milk intake values decreased from the beginning to the end of lactation (*p* < 0.001). Calf milk intake on d 25 was correlated with cow milk yield on d 23 (*r* = 0.47, *p* < 0.001), with cow and calf ADG during lactation (*r* = −0.53, *r* = 0.63, respectively, *p* < 0.001) and with calf LW at weaning (*r* = 0.74, *p* < 0.001). The BCS at calving influenced the calf milk intake on d 25 and 120 (*p* = 0.030), with 1.6 kg/d extra for each extra BCS point.

### 3.3. Immunoglobulin Concentration in Colostrum and Cow-Calf Plasma

**Figure 1:**
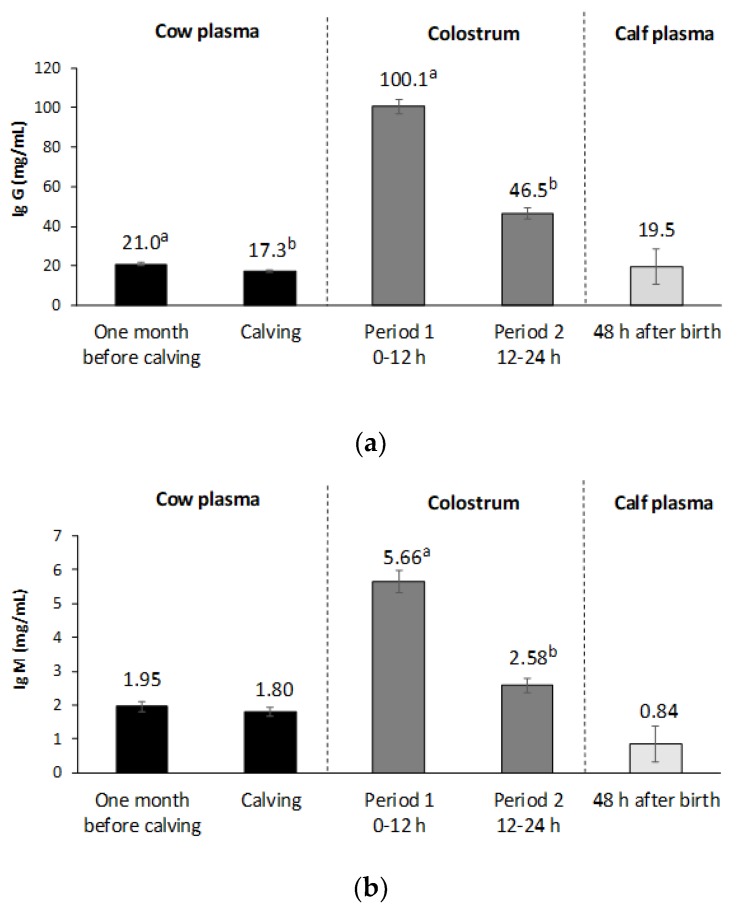
(**a**) Mean IgG concentrations in dam plasma, colostrum and calf plasma in the peripartum. (**b**) Mean IgM concentrations in dam plasma, colostrum and calf plasma in the peripartum. ^a,b^ Means with different superscripts between cow plasma or between colostrum samples differ significantly (*p* < 0.05).

The cow Ig concentration was not related to the nutritional treatment during early gestation or breed (*p* > 0.05). Plasma IgG concentration significantly decreased from 1 month before calving to calving by 17.7% (*p* < 0.001, Figure 1). The IgG concentration of colostrum during the first 12 h postpartum was six-fold higher than that from cow plasma at calving. From Period 1 to Period 2, colostrum IgG concentration decreased approximately 54% (*p* < 0.001). The colostrum IgG concentration in Period 1 was correlated with the calf ADG throughout lactation (*r* = 0.34, *p* = 0.002). An interaction was found between nutritional treatment and breed in average colostrum IgG concentration (between Period 1 and 2). No differences were found between CONTROL-PA and CONTROL-PI colostrum IgG concentration (74.1 ± 4.57 ng/mL vs. 76.0 ± 7.29 ng/mL, respectively, *p* > 0.05), but SUBNUT-PA had higher IgG values than SUBNUT-PI cows (82.16 ± 4.48 ng/mL vs. 62.1 ± 4.97 ng/mL, respectively, *p* < 0.004). In calf plasma IgG concentration, no nutritional treatment or breed effect was observed (*p* > 0.05). The calf plasma IgG concentration was higher (12.9%) than that obtained in cow plasma at calving and was correlated with calf ADG throughout lactation (*r* = 0.32, *p* = 0.022). Regarding IgM, cow plasma Ig concentration also decreased at the end of gestation but not significantly (*p* > 0.05). Colostrum IgM concentrations in Period 1 were three-fold higher than those obtained in cow plasma at calving, and from Period 1 to Period 2, its concentration halved (*p* < 0.001). Calf IgM concentration was not affected by maternal nutrition or breed (*p* > 0.05), and its value was lower (−53.3%) compared with dam plasma concentration.

### 3.4. Metabolic and Endocrine Profiles of Cows and Calves

**Figure 2:**
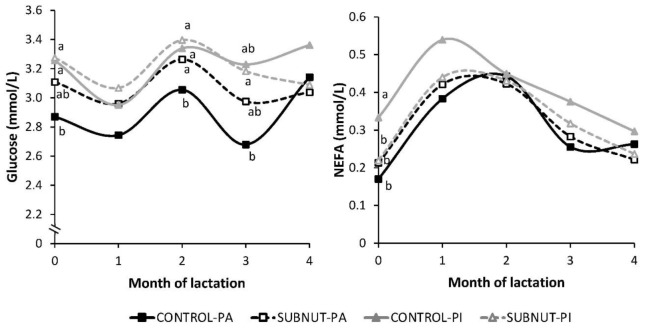
Cow metabolic profiles throughout lactation, according to the nutritional treatment and the breed. ^a,b^ Means within a month with different superscripts differ significantly (*p* < 0.05); CONTROL, 100% fed group; SUBNUT, 65% fed group; PA, Parda de Montaña; PI, Pirenaica.

**Figure 3:**
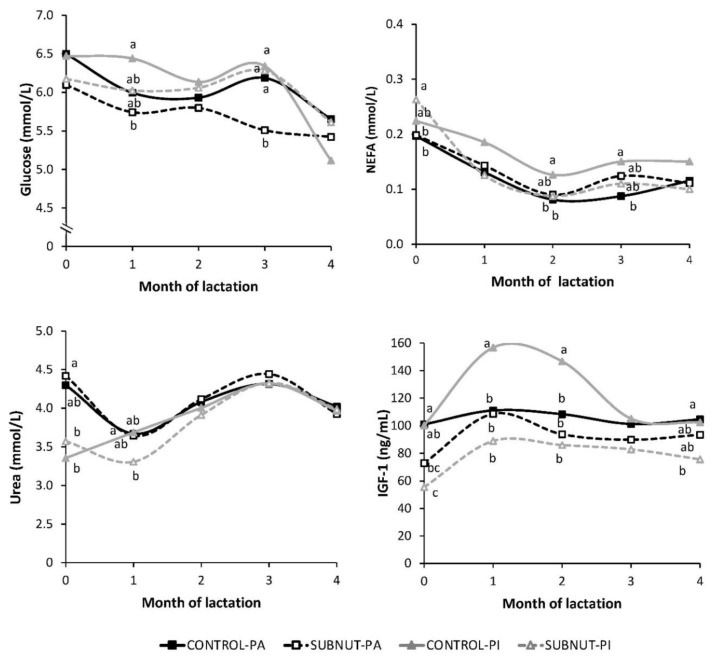
Calf metabolic and endocrine profiles throughout lactation, according to the nutritional treatment and the breed. ^a,b,c^ Means within a month with different superscripts differ significantly (*p* < 0.05); CONTROL, 100% fed group; SUBNUT, 65% fed group; PA, Parda de Montaña; PI, Pirenaica.

Metabolic and endocrine profiles are presented in Figure 2 (cows) and Figure 3 (calves). In cows, CONTROL-PA had the lowest plasma glucose concentrations throughout lactation, with statistical differences in SUBNUT-PA in month 2 (*p* < 0.05). Pirenaica cows had higher average glucose concentration than PA (3.2 ± 0.06 vs. 3.0 ± 0.04 mmol/L, respectively, *p* = 0.005). 

Regarding NEFA values, PI dams had higher average concentration than PA (0.36 ± 0.021 vs. 0.31 ± 0.014 mmol/L, respectively, *p* = 0.033). At calving, CONTROL-PI cows had the highest NEFA concentrations (*p* < 0.05).

In calves, glucose concentration was higher in CONTROL-PA than in SUBNUT-PA throughout lactation, with statistical differences in month 3 (*p* < 0.05). In general, CONTROL-PI had higher NEFA concentration than SUBNUT-PI, with statistical differences in month 2 (*p* < 0.05). Urea concentration was higher in PA than in PI calves at the beginning of the lactation, but from month 2, all animals had similar values. Regarding IGF-1 concentration, an interaction between nutritional treatment and breed was observed, because no statistical differences were found between CONTROL-PA and SUBNUT-PA average values during lactation (105.3 ± 5.83 ng/mL vs. 91.8 ± 5.54 ng/mL, respectively, *p* > 0.05), but CONTROL-PI had higher concentrations than SUBNUT-PI (122.3 ± 9.72 ng/mL vs. 77.8 ± 6.43 ng/mL, respectively, *p* < 0.001). Specifically, CONTROL-PI calves had higher values of IGF-1 than the other groups in month 2 and month 3 (*p* < 0.05). A correlation was found between the cow IGF-1 concentration in early gestation (d 28) and calf IGF-1 concentration at birth (*r* = 0.33, *p* = 0.003). Furthermore, calf IGF-1 concentration during lactation was also positively related to its ADG (*r* = 0.63, *p* < 0.001) and negatively with the cow ADG (*r* = −0.28, *p* = 0.011).

## 4. Discussion

### 4.1. Cow and Calf Performance

Poor nutrition during the first third of gestation had long-term effects on cow-calf performance. Although LW at calving was similar in CONTROL and SUBNUT cows, the BCS was lower in the SUBNUT group. This finding suggests that SUBNUT cows were not able to recover their body fat reserves due to the high energy demand in the last stages of gestation [23]. The discordance between BCS and LW shows that despite that these two variables have a strong relationship, the BCS reflects the body fat reserves of the animal, linked to its metabolic status, whereas LW is mainly linked to the animal dimensions, with a high hereditary component [24]. Furthermore, discordances between LW and BCS are common after calving due to changes in the carcass, digestive tract or visceral adipose tissue weight [25]. In our experiment, at the beginning of gestation, when the nutritional treatment started, the CONTROL and SUBNUT groups were BCS and LW balanced [13]. The BCS differences found at calving disappeared afterward, suggesting that when the high energy requirements from the end of gestation and the beginning of lactation decreased, the cows increased their BCS throughout lactation. By contrast, the cow ADG had negative values, with discordance between LW and BCS again. The BCS difference at calving between CONTROL and SUBNUT cows also affected calf performance, with large differences between CONTROL-PI and SUBNUT-PI calf LW at weaning but not at birth. To reduce bovine fetal growth, a severe nutritional restriction is required during at least the last half or third of the pregnancy [5,26,27]. The energy dietary restriction in early pregnancy could not influence calf birth weight, especially when cows received feed supplementation in the second half of gestation to ensure normal birth weight [28]. Although fetal size should not be affected by nutrition received during early and mid-pregnancy, placental characteristics may be altered [29,30]. In the current study, no differences were found in birth LW but were found at weaning; thus, fetal growth was adequate, but possibly due to some alterations in the fetal programming together with a low milk intake capacity, the correct postnatal calf development was altered [3]. Nutrition during early pregnancy could have more subtle effects on organ and tissue development, with potential long-term consequences, whereas nutrition during later pregnancy impacts on fetal and carcass growth most [26]. In the first third of gestation, SUBNUT cows had a negative energy balance, which means that the fetus had to adapt its metabolism to a poor uterine environment. In most of the cases, these fetal adaptive mechanisms were irreversible with consequences in postnatal life [31], highlighting the crucial role of maternal nutrition during the first stages of gestation. 

Regarding the breed, PI had higher BCS during lactation and lower calf LW at birth than PA, in concordance with the results reported by Sanz et al. [32] and Rodríguez-Sánchez et al. [12]. The differences found in ADG between CONTROL-PI and SUBNUT-PI calves indicate that PI breed is more sensitive to undernourishment than PA, in line with the results of Noya et al. [13], with greater long-term detriment on its offspring postnatal development.

The morphometric measurements indicated a faster body growth of CONTROL calves throughout lactation. No differences were found in the first week of life; however, CONTROL calves were larger than SUBNUT calves at weaning in most of the parameters registered. The fetus intrauterine growth in the SUBNUT group seemed to be adequate, with similar LW at birth between groups, but during lactation, the CONTROL group grew faster than the SUBNUT group. The breed influenced some body measurements in the first week of life, at weaning, or both, proving the inter-breed morphology and development differences.

The BCS at calving had a substantial influence on the resumption of cow ovarian cyclicity. The CONTROL group required fewer days to recover its ovarian activity, although the difference was not significant. Regarding the breed, the PI cows had a shorter postpartum anoestrus length, related to their higher BCS at calving [33]. Sanz et al. [34] reported no differences in the postpartum anoestrus length between PA and PI cows, confirming our hypothesis that the breed difference found in our study was attributed to a BCS effect. The degree of the negative energy balance that the cows undergo in early lactation modifies the time needed for the first ovulation. During a negative energy balance, luteinizing hormone pulses are suppressed and dominant follicles that develop have a lower chance of producing a sufficient amount of estradiol to induce a pre-ovulatory gonadotrophin surge [35].

### 4.2. Colostrum and Milk Composition, Milk Yield and Calf Milk Intake

The nutritional treatment applied during early gestation did not affect the colostrum chemical composition. Similarly, Quigley and Drewry [23] found that the manipulation of cow diet to alter the fat content of colostrum did not result in increased colostrum energy content. Neither the breed nor BCS at calving had any effect on its quality, in concordance with Morrill et al. [36]. Colostrum has an extremely high protein concentration; however, the protein content declines sharply a few hours after calving because of the Ig transfer ceases from the dam’s circulation to the mammary secretions [37]. Thus, in this study, the protein content decreased significantly from Period 1 to Period 2, and the lactose content increased.

After calving, the duration of the transition from colostrum to milk can last between two and seven days [38,39], during which the lactose content increases and the fat, protein and somatic cell count values decrease [40]. However, other authors have reported that the fat content in mature milk can also increase [41], as observed in our results. In our experiment, in the transition from colostrum to milk, the somatic cell counts decreased drastically. The high colostrum somatic cell counts were not related to a mastitic infection but a high temporary permeability of tight junctions between the mammary epithelial cells [42]. 

The milk fat content on d 23 was higher in SUBNUT than in CONTROL cows. This may be associated with the higher milk yield of CONTROL than SUBNUT cows, despite the fact that it was not significant. Milk yield and fat percentage are negatively correlated [43], because an increase in milk yield implies a dilution effect in fat milk constituents [44]. Inter-breed differences were supported by our results because PA cows had higher milk yield with lower fat and lactose content than PI, in accordance with the results reported by Álvarez-Rodríguez et al. [18]. Both in colostrum and milk, PA cows had higher somatic cell counts than PI, which can be associated with their higher milk yield [45].

An interaction was observed here between breed and maternal nutritional treatment in calf milk intake on d 25, which was lower in SUBNUT-PI calves than in the others, although the milk yield of SUBNUT-PI cows on d 23 was similar to that of their CONTROL-PI counterparts. These differences may be due to a lesser digestive tract development of SUBNUT-PI calves, either during fetal life or immediately after birth, which impaired their milk intake capacity. The calf milk intake at the beginning of lactation could be a good indicator of the calf LW at weaning, but a moderately accurate method to estimate the cow milk yield in suckler cows. Calf milk intake on d 25 had a positive relationship with calf ADG during lactation and a negative relationship with cow ADG. Animals with higher milk aptitude, such as PA, prioritize the allocation of dietary energy to the milk yield, increasing calf ADG values, instead of their own weight gains. By contrast, lactating PI dams prioritized their own maintenance over milk production and the development of their offspring, accordingly with the results obtained by Sanz et al. [32].

### 4.3. Immunoglobulin Concentration in Colostrum and Cow-Calf Plasma

Plasma concentrations of IgG and IgM in cows were not influenced by the nutritional treatment during early gestation or the breed. They decreased in the last month of gestation, although not significantly in the case of IgM. The reduction of plasma Ig in the third trimester of gestation is a physiological phenomenon: Ig are transferred from a dam’s circulation into the udder tissue [46]. Plasma IgG drop is described from the eighth week before calving, whereas IgM concentration starts to decrease from the fourth week before calving [47].

Colostrogenesis occurs as early as five weeks before calving [48], suggesting that the undernutrition applied during the first third of gestation should not affect the colostrum Ig concentration [49]. However, the lower BCS at calving of SUBNUT cows could indirectly affect the colostrum Ig concentration. In our study, colostrum IgG concentration was higher in SUBNUT-PA than in SUBNUT-PI dams, supporting the hypothesis that PI could be more sensitive to negative energy balance.

In this study, IgG and IgM concentration in colostrum decreased dramatically from Period one to Period two because of the cessation of the Ig transfer immediately before calving, in accordance with the results reported by Barrington and Parish [37]. Notably, colostrum Ig concentration was negatively associated with the interval from calving to colostrum collection [50]. 

Our findings did not support the hypothesis that maternal undernutrition could adversely impact the passive transfer of immunity from dam to calf. Despite the lower colostrum IgG concentration in SUBNUT-PI cows, no differences between nutritional treatments or breeds were observed in IgG and M plasma concentrations in calves, in accordance with studies that have described no adverse impact of maternal dietary restriction on calf passive immunity [10,49]. By contrast, Burton et al. [51] found that the absorption of IgG1, IgG2, IgM, and IgA were reduced in calves born from nutrient-restricted dams, but the colostrum Ig concentration was not affected. Hammer et al. [52] described higher IgG plasmatic values in lambs whose mothers were nutrient restricted during pregnancy, suggesting that the fetal gastrointestinal system may be programmed to be more efficient in extracting nutrients, namely, large molecules such as Ig. In the current study, IgG concentration in colostrum and calf plasma was correlated with calf ADG. Wittum and Perino [53] described an indirect effect of passive transfer status on calf ADG and weaning weight because of its effect on calf morbidity. By contrast, Cummins et al. [54] reported no differences in daily weight gains during lactation in calves with different plasma IgG concentrations 24 h after birth.

### 4.4. Metabolic and Endocrine Profiles of Cows and Calves

The cow glucose profiles were related to inter-breed differences, with higher concentrations in PI cows, and to the current diet received during lactation, instead of the nutritional treatment applied during early pregnancy. Glucose profiles are strongly linked with the short-term effect of the current energy and/or protein intake [12]. 

The BCS difference at calving due to undernutrition in early pregnancy was reflected in NEFA profiles, specifically in PI cows. Non-esterified fatty acids concentrations were 50% higher in CONTROL-PI than in SUBNUT-PI cows. During early lactation, an imbalance is observed between the high energy requirements and the reduced intake capacity [35], and cows mobilize body fat reserves as an energy source, increasing the plasma NEFA concentrations [55] and leading to LW losses. In this study, CONTROL-PI cows mobilized more lipid stores, increasing their plasma NEFA concentration. Non-esterified fatty acids concentration in all groups was higher between month one and month two of lactation, by the time the peak milk yield is attained, with the greatest nutritional demands [18].

Regarding calf metabolic profiles, glucose concentration was higher than that obtained in cows, accordingly with the calf diet [56]. Glucose is sourced from milk and, furthermore, during the first month of life, glucose intake is insufficient to maintain the normal plasma glucose concentration, and liver gluconeogenesis is activated [57]. In this study, early maternal nutrition had little impact on calf glucose metabolism. Similarly, other studies have described no effect of nutrient restriction during early gestation on calf glucose basal concentration [58,59]. 

Non-esterified fatty acids concentration in calves decreased at the beginning of lactation with a nadir in month 2, probably due to the peak milk yield of cows. After calving, calves moved from an intrauterine diet comprising primarily glucose and amino acids to a diet higher in fat [26], with increased energy content that reduced the high plasmatic NEFA concentration observed in the first days of life. From month 2, CONTROL-PI calves had higher values than their counterparts, which could reflect a higher amount and turnover of adipose tissue.

Urea values were not related to maternal nutrition in early pregnancy but to calf nutrition, reflecting its dependence on current energy and protein intake [60]. Blood urea concentration is influenced by the degree of protein catabolism and the ratio of energy to protein in the diet. Some authors have also described a plasma urea increase in newborns after a high amount of colostrum intake due to its high protein concentration [57]. 

Maternal nutrition affected calf plasma IGF-1 concentration. In general, CONTROL calves had higher IGF-1 concentrations than SUBNUT calves. These differences between nutritional treatment groups were higher in PI breed, supporting that the long-term effects of maternal subnutrition are greater in PI than in the PA breed, in accordance with results reported by Noya et al. [13]. The IGF-1 concentration was correlated with calf ADG and reflected in the lower LW of SUBNUT-PI calves at weaning. The IGF-1 is critical in the control of animal growth, and greater serum IGF-1 concentration is associated with faster growth rates [61]. In newborns, the growth hormone-IGF-1 axis is already functional, and the still low plasma IGF-1 concentration increases approximately 2.5-fold up to nine months of age [61]. In this study, poor maternal nutrition during the first third of pregnancy could have reprogrammed the fetal IGF-1 system in its ability to respond to acute changes in the substrate supply [62], resulting in evidence of different calf IGF-1 concentrations at birth, when the cow performance would still have little impact on calf metabolic and endocrine profiles. Our results imply a transgenerational relationship between IGF-1 concentrations of cows during early gestation, which were conditioned by the cow feeding level, with the IGF-1 values of their progeny at birth. Similarly, Maresca et al. [63] described lower calf IGF-1 concentration at birth after maternal protein intake restriction from mid-gestation to calving. In our study, although these IGF-1 differences decreased at the end of lactation, SUBNUT calves had reduced LW and body measurements at weaning, reflecting a residual effect of maternal undernutrition on offspring postnatal growth. 

## 5. Conclusions

In this study, the undernutrition applied during the first third of gestation in suckler cows had long-term effects on the productive efficiency of the cow-calf pair in the following lactation. Undernourished cows had a lower BCS at calving, which impaired most of the studied cow-calf traits. Additionally, feed restriction could have modified the calf fetal programming, which was reflected in their lower performance and IGF-1 concentration after birth. These long-term effects, associated with a reduced calf milk intake, impaired body growth and LW at weaning of SUBNUT calves, these effects being more severe in Pirenaica cow-calf pairs. Further research should investigate the impact of early maternal subnutrition on the post-weaning efficiency of beef heifers and young bulls.

## Figures and Tables

**Table 1:** Chemical composition of feedstuffs used in the experiment (on an as-fed basis).

Chemical Composition	
DM (g/kg)	908
CP (g/kg DM)	124
NDF (g/kg DM)	466
ADF (g/kg DM)	253
ADL (g/kg DM)	40
Ash (g/kg DM)	113
ME (MJ/kg DM)	10.96

DM, dry matter; CP, crude protein; NDF, neutral detergent fiber; ADF, acid detergent fiber; ADL, acid-detergent lignin; ME, metabolizable energy.

**Table 2:** Live weight (LW) and body condition score (BCS) of cows throughout lactation, according to the nutritional treatment and the breed.

Traits	Nutritional Treatment		Breed		RSD	Significance	
	CONTROL	SUBNUT	PA	PI		Nut. Treat.	Breed
**Cow LW (kg)**							
At calving	605	592	598	599	54.4	n.s.	n.s.
At weaning	586	576	575	588	53.5	n.s.	n.s.
**BCS (1–5**							
At calving	2.8 ^a^	2.7 ^b^	2.7 ^b^	2.9 ^a^	0.22	0.032	<0.001
At mid lactation	2.9	2.8	2.7 ^b^	3.0 ^a^	0.26	n.s.	<0.001
At weaning	3.0	3.0	2.8 ^b^	3.1 ^a^	0.28	n.s.	<0.001

^a,b^ Means within a row with different superscripts differ significantly (*p* < 0.05); n.s., not significant (*p* > 0.05); CONTROL, 100% fed group; SUBNUT, 65% fed group; PA, Parda de Montaña; PI, Pirenaica; RSD, residual standard deviation.

**Table 3:** Calf live weight (LW) and average daily gain (ADG) of both cows and calves throughout lactation, according to the interaction between nutritional treatment and breed.

Traits	Nutritional Treatment × Breed				RSD	Significance
	CONTROL-PA	SUBNUT-PA	CONTROL-PI	SUBNUT-PI		
**Calf LW (kg)**						
At birth	45 ^a^	46 ^a^	39 ^b^	40 ^b^	5.9	0.002
At weaning	149 ^a^	146 ^a^	155 ^a^	126 ^b^	19.9	<0.001
**ADG (kg/d)**						
Cows	−0.151 ^b^	−0.188 ^b^	−0.179 ^b^	−0.004 ^a^	0.1742	0.014
Calves	0.807 ^a^	0.792 ^a^	0.860 ^a^	0.672 ^b^	0.1582	0.042

^a,b^ Means within a row with different superscripts differ significantly (*p* < 0.05); CONTROL, 100% fed group; SUBNUT, 65% fed group; PA, Parda de Montaña; PI, Pirenaica; RSD, residual standard deviation.

**Table 4:** Calf morphometric measurements throughout lactation, according to the nutritional treatment and the breed (cow BCS at calving included as a covariate).

Traits	Nutritional Treatment		Breed		RSD	Significance		
	CONTROL	SUBNUT	PA	PI		Nut. Treat.	Breed	BCSc
**Height at withers (cm)**								
d 7	74	73	75 ^a^	72 ^b^	3.4	n.s.	0.001	n.s.
d 120	94 ^a^	93 ^b^	95 ^a^	92 ^b^	3.2	0.046	<0.001	n.s.
**Height at rump (cm)**								
d 7	78	78	79	77	3.7	n.s.	n.s.	n.s.
d 120	101	99	101 ^a^	98 ^b^	4.0	n.s.	0.002	n.s.
**Rump width (cm)**								
d 7	18	18	19 ^a^	18 ^b^	1.9	n.s.	0.045	n.s.
d 120	26	26	26	26	2.4	n.s.	n.s.	n.s.
**Rump length (cm)**								
d 7	21	21	22	21	1.8	n.s.	n.s.	n.s.
d 120	35 ^a^	34 ^b^	34	35	2.8	0.020	n.s.	n.s.
**Body length (cm)**								
d 7	67	66	67	66	3.9	n.s.	n.s.	n.s.
d 120	97 ^a^	95 ^b^	96	96	4.9	0.022	n.s.	n.s.
**Heart girth (cm)**								
d 7	86	84	88 ^a^	83 ^b^	5.1	n.s.	<0.001	<0.001
d 120	119 ^a^	115 ^b^	118 ^a^	115 ^b^	5.5	0.004	0.024	<0.001

^a,b^ Means within a row with different superscripts differ significantly (*p* < 0.05); n.s., not significant (*p* > 0.05); CONTROL, 100% fed group; SUBNUT, 65% fed group; PA, Parda de Montaña; PI, Pirenaica; RSD, residual standard deviation; BCSc, cow body condition score at calving.

**Table 5:** Colostrum composition, according to the nutritional treatment and the breed (cow BCS at calving included as a covariate).

Traits	Nutritional Treatment		Breed		RSD	Significance		
	CONTROL	SUBNUT	PA	PI		Nut. Treat.	Breed	BCSc
**Fat (%)**								
Period 1	3.5	3.2	3.5	3.2	1.83	n.s.	n.s.	n.s.
Period 2	3.3	3.9	3.3	4.0	2.34	n.s.	n.s.	n.s.
**Protein (%)**								
Period 1	17.5 ^y^	18.3 ^y^	18.1 ^y^	17.7 ^y^	2.85	n.s.	n.s.	n.s.
Period 2	11.6 ^z^	11.3 ^z^	11.0 ^z^	12.0 ^z^	3.07	n.s.	n.s.	n.s.
**Lactose (%)**								
Period 1	3.2 ^z^	3.1 ^z^	3.0 ^b,z^	3.3 ^a,z^	0.49	n.s.	0.015	n.s.
Period 2	3.5 ^y^	3.5 ^y^	3.4 ^y^	3.6 ^y^	0.55	n.s.	n.s.	n.s.
**Somatic cell count (n × 10^3^/mL)**								
Period 1	1276	1043	1526 ^a^	872 ^b^	-	n.s.	0.029	<0.001
Period 2	1464	1510	1890	1170	-	n.s.	n.s.	<0.001

^a,b^ Means within a row with different superscripts differ significantly (*p* < 0.05); ^y,z^ Means within a column and trait with different superscripts differ significantly by period (*p* < 0.05); n.s., not significant (*p* > 0.05); CONTROL, 100% fed group; SUBNUT, 65% fed group; PA, Parda de Montaña; PI, Pirenaica; Period 1, from 0 to 12 h postpartum; Period 2, from 12 and 24 h postpartum; RSD, residual standard deviation; BCSc, cow body condition score at calving.

**Table 6:** Cow milk yield and composition, and calf milk intake, according to the nutritional treatment, the breed or their interaction (cow BCS at calving included as a covariate).

Traits	Nutritional Treatment		Breed		RSD	Significance		
	CONTROL	SUBNUT	PA	PI		Nut. Treat.	Breed	BCSc
Milk yield d 23 (kg/d)	9.5	8.7	9.9 ^a^	8.3 ^b^	2.11	n.s.	0.008	0.049
Energy-corrected milk yield (kg/d)	10.2	9.6	10.0	9.7	2.20	n.s.	n.s.	0.049
Fat (%)	4.4 ^b^	4.8 ^a^	4.2 ^b^	4.9 ^a^	0.60	0.010	<0.001	0.016
Protein (%)	3.6	3.7	3.6	3.7	0.30	n.s.	n.s.	0.030
Lactose (%)	4.7	4.7	4.6 ^b^	4.9 ^a^	0.29	n.s.	<0.001	n.s.
Somatic cell count (n × 10^3^/mL)	184	174	314.4 ^a^	102.1 ^b^	-	n.s.	0.002	n.s.
**Traits**	**Nutritional treatment × Breed**					**RSD**	**Significance**	
	**CONTROL-PA**	**SUBNUT-PA**	**CONTROL-PI**	**SUBNUT-PI**			**N.T. × Breed**	**BCSc**
Calf milk intake (kg/d)								
d 25	9.4 ^a^	9.0 ^a^	8.2 ^a^	6.7 ^b^		1.53	<0.001	0.030
d 120	7.5 ^a^	7.5 ^a^	5.6 ^b^	5.1 ^b^		1.93	<0.001	0.030

^a,b^ Means within a row with different superscripts differ significantly (*p* < 0.05); n.s., not significant (*p* > 0.05); CONTROL, 100% fed group; SUBNUT, 65% fed group; PA, Parda de Montaña; PI, Pirenaica; RSD, residual standard deviation; N.T., nutritional treatment; BCSc, cow body condition score at calving.
